# Use of Pulmonary Artery Catheter in Coronary Artery Bypass Graft. Costs and Long-Term Outcomes

**DOI:** 10.1371/journal.pone.0117610

**Published:** 2015-02-17

**Authors:** Fei Xu, Qian Wang, Heng Zhang, Sipeng Chen, Hushan Ao

**Affiliations:** 1 Department of Anesthesiology, Cardiovascular Institute and Fuwai Hospital, Chinese Academy of Medical Sciences, Beijing, China; 2 Department of Anesthesiology, Inner Mongolia Medical University, Huhhot Inner Mongolia, China; 3 Department of cardio-thoracic surgery, Cardiovascular Institute and Fuwai Hospital, Chinese Academy of Medical Sciences, Beijing, China; 4 Department of Biostatistical Unit, Capital Medical University, Beijing, China; Thomas Jefferson University, UNITED STATES

## Abstract

**Background:**

Pulmonary artery catheters (PAC) are used widely to monitor hemodynamics in patients undergoing coronary bypass graft (CABG) surgery. However, recent studies have raised concerns regarding both the effectiveness and safety of PAC. Therefore, our aim was to determine the effects of the use of PAC on the short- and long-term health and economic outcomes of patients undergoing CABG.

**Methods:**

1361 Chinese patients who consecutively underwent isolated, primary CABG at the Cardiovascular Institute of Fuwai Hospital from June 1, 2012 to December 31, 2012 were included in this study. Of all the patients, 453 received PAC during operation (PAC group) and 908 received no PAC therapy (control group). Short-term and long-term mortality and major complications were analyzed with multivariate regression analysis and propensity score matched-pair analysis was used to yield two well-matched groups for further comparison.

**Results:**

The patients who were managed with PAC more often received intraoperative vasoactive drugs dopamine (70.9% vs. 45.5%; P<0.001) and epinephrine (7.7% vs. 2.6%; P<0.001). In addition, costs for initial hospitalization were higher for PAC patients ($14,535 vs. $13,873, respectively, p = 0.004). PAC use was neither associated with the perioperative mortality or major complications, nor was it associated with long-term mortality and major adverse cardiac and cerebrovascular events. In addition, comparison between two well-matched groups showed no significant differences either in baseline characteristics or in short-term and long-term outcomes.

**Conclusions:**

There is no clear indication of any benefit or harm in managing CABG patients with PAC. However, use of PAC in CABG is more expensive. That is, PAC use increased costs without benefit and thus appears unjustified for routine use in CABG surgery.

## Introduction

Since the introduction of pulmonary artery catheter (PAC) in 1970s, PAC was used significantly as hemodynamic monitoring device in clinical practice [1]. For patients who undergo coronary artery bypass graft (CABG) surgery, the PAC remains the most frequently used monitor among cardiovascular anesthesiologists [2]. The PAC has been considered to a valuable device in perioperative fluid and vasoactive drug managements. It is clear that the PAC provides hemodynamic data that are used to make therapeutic decisions. The critical question is whether these therapeutic decisions improve outcomes.

Several randomized controlled trials (RCT) have reported no benefits following PAC insertion [3–5]. However, these studies were limited by their small size and lacked strictly defined treatment protocols. Because no large randomized studies are currently planned to evaluate the outcomes or costs for PAC use in CABG surgery, clinicians must rely on observational data to make decisions regarding the optimal use of PAC in this setting [6]. We analyzed records of patients who underwent CABG surgery and were managed with or without perioperative pulmonary artery catheterization to clarify the clinical effects of PAC use.

## Materials and Methods

### Study population

This was a retrospective, observational study of consecutive patients who underwent isolated primary CABG at the Fuwai Hospital. The study protocol was approved by the Ethics Committee of Fuwai Hospital, and written informed consent was waived. 1361 patients who consecutively underwent isolated, primary CABG from June 1, 2012 to December 31, 2012 at Fuwai Hospital in Beijing, China were included in this study: 453 received a PAC during surgery (PAC group) and 908 received no PAC therapy (control group). All the extracted patients were ethnic Chinese. Isolated primary CABG was defined as coronary artery bypass graft surgery alone for the first time with or without cardiopulmonary bypass. Patients who underwent combined cardiac surgical procedures were excluded. The data were collected throughout hospitalization materials and follow-up data. Follow-up was done through telephone, letters and callback examinations.

### PAC Definition and Management

Perioperative PAC use was at the discretion of the anesthesiologist and was not randomized. The patients were divided into the PAC or non-PAC group according to the presence of a PAC after central venous catheter insertion. Intraoperative anesthesia management was consistent among our cardiac anesthesiologists with an institutional standard of a moderate dose of narcotic (fentanyl or sufentanil) supplemented by a volatile anesthetic agent.

Although institutional preferences were for PAC monitoring when left ventricle function was impaired for CABG patients as determined by preoperative transthoracic echocardiogram, the decision about intraoperative PAC use rested solely on the attending anesthesiologist’s preference. All anesthesiologists involved in the study were experienced cardiac anesthesiologists. For CABG patients, the anesthesiologists decide whether to insert a PAC or not depending on various factors that include patients’ EF, left ventricular end-diastolic diameter, presence of ventricular aneurvsm, and after evaluating left ventricular function. In addition, preoperative myocardial infarction, pulmonary artery hypertension, and preoperative intra-aortic balloon pump, and so on also are involved. With these factors anesthesiologists will incorporate their own experience to guide PAC application. The PAC or non-PAC hemodynamic diagnoses and treatments were not controlled or standardized. The patients were managed according to individual practice patterns, and the clinical decisions were not dictated by a study protocol.

### Measurement of Outcomes

All outcomes were specified prior to analysis and were defined by the protocol. In-hospital mortality was defined as death during the primary hospitalization. Myocardial infarction was defined as the presence of new Q waves in two or more contiguous leads on the electrocardiogram [7]. Cerebrovascular accidents were defined as central neurologic deficits that persisted for more than 72 h [8]. Renal failure was defined as the need for dialysis to treat prolonged oliguria or anuria [9].

### Cost data

In-hospital costs were obtained directly from the medical records and included diagnostic, procedural, and post-procedural costs. The costs per patient were calculated by multiplying the resources used by the unit costs. We used the diagnostic-related group price in the Medicare claims data from the Beijing Medical Insurance Center for the unit cost estimates. Medication costs were based on the drug type, dosage, and route of administration, as reported by the patient, and subsequently multiplied by drug prices at our institution. The costs were adjusted to 2012 Ren Min Bi (RMB) with the Beijing consumer price index of the Chinese National Bureau of Statistics (http://www.stats.gov.cn).

### Statistical Analysis

Baseline data are presented as mean±SD continuous variables, and as frequencies and percentages for categorical variables. Continuous variables were compared using t tests, categorical variables using χ^2^ tests. In-hospital outcomes were compared using multiple logistic regression models with the outcomes measure as the dependent variable and patient characteristics and operative variables as independent variables. Kaplan-Meier curves and log rank tests were used to compare long-term survival between groups. Hazard ratios(HR) were estimated by the Cox proportional hazards regression models. We examined the differential effect of two groups after adjusting for covariates. Confounders were included into the multivariable logistic and Cox regression analyses if the p value equal or less than 0.05. Cost data are reported as both mean±SD, and compared comparisons were performed by t tests.

In addition, we compared outcomes by propensity-score matching to yield well-matched pairs. The matched-pairs analysis was performed according to the propensity score for the 453 patients in the control group. They were matched in a one-to-one ratio to patients who received PAC therapy.

All reported *P*-values are two-sided and *P*-values ≤0.05 were considered to indicate statistical significance. All statistical analyses were performed using SAS 9.4 (SAS Institute Inc., Cary, NC, USA)

## Results

### Baseline Characteristics

Table 1 shows patient demographic data. From the 1361 eligible patients, there were no significant differences between the two groups with respect to diabetes mellitus, hypertension, hyperlipidemia, smoking, drinking, history of renal failure, COPD, peripheral vascular disease, cerebral events, pulmonary arterial hypertension. However, the patients in the PAC group were older (62.5±9.1 vs. 60.5±8.5); had higher BMI(25.2±3.2 vs. 25.6±3.0); more frequent chronic liver disease (3.1% vs. 0.8%), myocardial infarction (56.1% vs. 27.4%), intra-aortic balloon pump use (2.1% vs.0.2%), ventricular aneurysm (8.6% vs. 0.3%), pulmonary arterial hypertension (1.8% vs. 0.6%), ejection fraction (55.9±9.3 vs. 62.6±10.8), left ventricular end-diastolic diameter (51.7±5.7 vs. 49.8±7.0). In other words, the condition of patients in the PAC group was relatively more severe than those in the control group. No significant differences in baseline characteristics between propensity-matched groups were observed.

**Table 1:** Baseline Characteristics.

Characteristics	Entire cohort		P value	Propensity-matched cohort		P value
	PAC	No PAC		PAC	No PAC	
	N = 453	N = 908		N = 424	N = 424	
Age (year)	62.5±9.1	60.5±8.5	<0.001	62.4±9.2	63.1±7.8	0.241
Female sex, n (%)	122(26.9)	198(21.8)	0.036	114(26.9)	90(21.2)	0.054
BMI	25.2±3.2	25.6±2.96	0.032	25.3±3.290	25.5±3.0	0.214
Medical history, n (%)						
Diabetes mellitus	145(32.0)	280(30.8)	0.660	136(32.1)	121(28.5)	0.191
Hypertension	290(64.0)	609(67.1)	0.262	271(63.9)	256(60.4)	0.288
Hyperlipidemia	284(62.7)	585(64.4)	0.530	266(62.7)	249(58.7)	0.232
Regular smoking	263(58.1)	509(56.1)	0.483	246(58.0)	245(57.8)	0.945
Drinking	80(17.7)	161(17.7)	0.974	75(17.7)	74(17.5)	0.928
History of renal failure	5(1.1)	14(1.5)	0.516	5(1.2)	9(2.1)	0.281
Chronic liver disease	14(3.1)	7(0.8)	0.001	13(3.1)	8(1.9)	0.269
COPD	14(3.1)	17(1.9)	0.156	13(3.1)	21(5.0)	0.161
Peripheral vascular disease	22(4.9)	41(4.5)	0.778	21(5.0)	22(5.2)	0.876
Cerebral events	44(9.7)	73(8.0)	0.299	41(9.7)	35(8.3)	0.471
Myocardial infarction	254(56.1)	249(27.4)	<0.001	238(56.1)	175(41.3)	0.449
Chronic heart failure	3(0.7)	1(0.1)	0.076	3(0.7)	1(0.2)	0.316
Atrial fibrillation	12(2.7)	19(2.1)	0.517	11(2.6)	9(2.2)	0.651
Intra-aortic balloon pump	19(2.1)	2(0.2)	<0.001	8(1.9)	2(0.5)	0.056
Three-vessel disease	377(83.2)	769(84.7)	0.484	353(83.3)	336(79.3)	0.135
Main stem disease	142(31.4)	251(27.6)	0.155	133(31.4)	109(25.7)	0.068
Ventricular aneurysm	39(8.6)	3(0.3)	<0.001	10(2.4)	3(0.7)	0.051
Pulmonary arterial hypertension	8(1.8)	5(0.6)	0.030	8(1.9)	5(1.2)	0.402
Ejection fraction	55.9±9.3	62.6±10.8	<0.001	57.6±8.2	58.6±8.3	0.085
LVEDD	51.7±5.7	49.8±7.0	<0.001	51.1±5.7	51.3±7.9	0.705

BMI indicates body mass index; COPD, chronic obstructive pulmonary disease; LVEDD, left ventricular end-diastolic diameter. Values are n(%) for categorical variables and mean±SD for continuous variables.

### In-hospital Outcomes

Table 2 illustrates the use of intraoperative vasoactive drugs and post-operative clinical outcomes between two groups. The patients in PAC group were received more dopamine (70.9% vs. 45.5%; P<0.001) and epinephrine (7.7% vs. 2.6%; P<0.001) than were those in the control group.

**Table 2:** Intraoperative Vasoactive Drugs and Postoperative Outcomes.

Variable	Entire cohort		Adjusted OR	95% CI	P Value	Propensity-matched cohort		P Value
	PAC	Non-PAC				PAC	Non-PAC	
	N = 453	N = 908				N = 424	N = 424	
Nitroglycerin	183(40.40)	326(35.90)	0.732	0.274–1.518	0.451	164(38.60)	148(35.09)	0.255
Dopamine	321(70.86)	413(45.48)	2.923	2.267–3.770	<0.001	309(72.88)	192(45.28)	<0.001
Epinephrine	35(7.73)	24(2.64)	2.796	1.623–3.816	<0.001	33(7.78)	13(3.07)	0.002
In-hospital death	6(1.32)	10(1.10)	0.636	0.170–2.384	0.502	6(1.42)	5(1.18)	0.762
Myocardial infarction	3(0.66)	4(0.44)	1.464	0.337–6.350	0.611	3(0.71)	2(0.47)	0.654
Atrial fibrillation	41(9.05)	78(7.95)	2.156	0.755–6.153	0.151	38(8.96)	33(7.78)	0.535
Cerebrovascular accident	3(0.66)	4(0.44)	0.432	0.074–2.518	0.351	3(0.71)	3(0.71)	NA
Acute renal failure	10(2.21)	18(1.83)	1.894	0.625–5.741	0.259	9(2.12)	7(1.65)	0.614
Reoperation for bleeding	7(1.54)	11(1.12)	0.892	0.288–2.758	0.843	7(1.65)	6(1.42)	0.780
Infective complications	4(0.88)	7(0.77)	0.968	0.924–1.105	0.184	4(0.94)	3(0.71)	0.704

OR, odds ratios; CI, confidence interval.

In-hospital mortality was 1.2% in the overall cohort, 1.3% in the PAC group, and 1.1% in the control group. There was no difference in in-hospital mortality (adjusted odds ratios [OR] 0.636, 95% confidence interval [CI]: 0.170 to 1.384). After adjusting for confounders, multivariable logistic regression analysis confirmed that PAC was not an independent risk predictor for those perioperative adverse outcome events or in-hospital death.

### Long-term Outcomes

**Fig 1:**
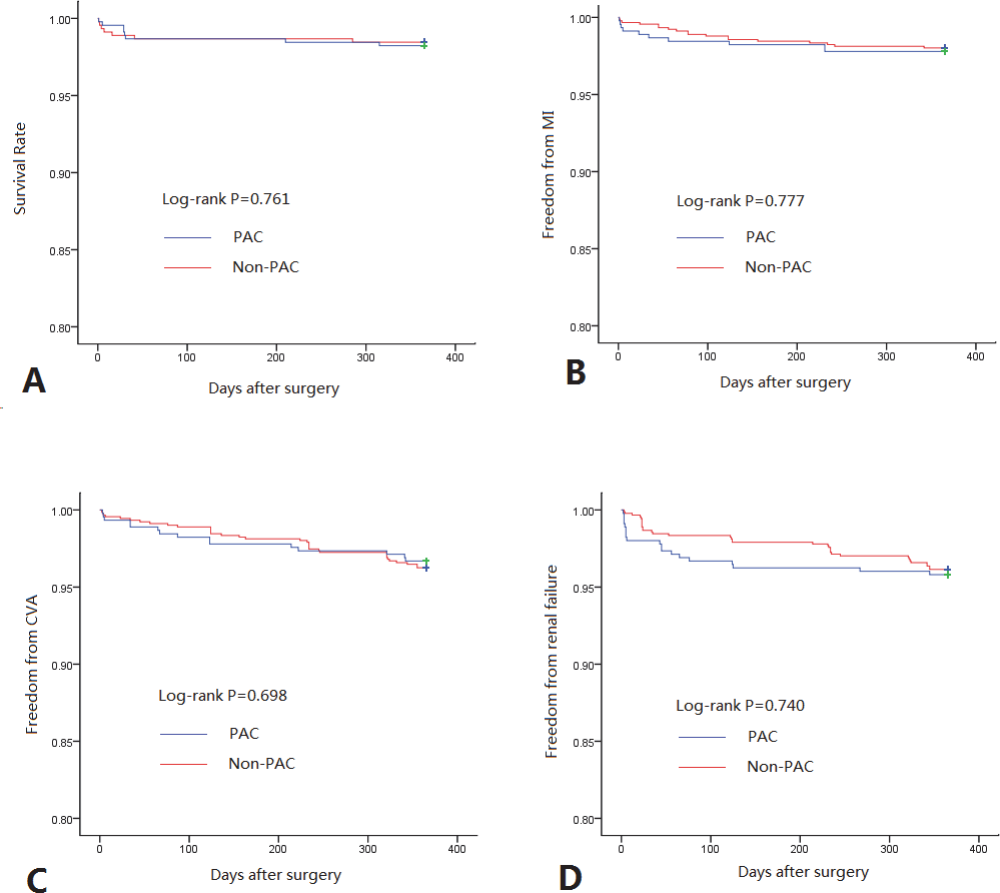
Long-Term Outcomes according to PAC status. A. Survival rate B. Freedom from myocardial infarction (MI) C. Freedom from cerebrovascular accident (CVA) D. Freedom from renal failure.

Of the 1361 patients, follow-up data were complete for 97.1%, during which period 22 patients died. In the multivariable Cox regression analysis, use of PAC was not associated with the risk of the long-term mortality (adjusted hazard ratio [HR] 1.135, 95% confidence interval [CI]: 0.371 to 2.470). There were also no significant differences in myocardial infarction, cerebrovascular accident and renal failure. Fig. 1 showed the Kaplan-Meier event-free survival analysis for long-term mortality, myocardial infarction, cerebrovascular accident and renal failure. The log-rank test was used and indicated no significant difference either in mortality or complications between the two groups.

### Propensity-Matched Groups and Outcomes

As a result of propensity matching, the derived two groups included 424 patients, and were well-matched and showed no difference in baseline characteristics (Table 1). Further comparison between the two well-matched groups showed no significant difference in either short- (Table 2) or long-term outcomes (Table 3) as mentioned in the above analysis.

**Table 3:** Long-term Outcomes.

Variable	Entire cohort		Adjusted HR	95% CI	P Value	Propensity-matched cohort		P Value
	PAC	Non-PAC				PAC	Non-PAC	
	N = 453	N = 908				N = 424	N = 424	
Death	8(1.77)	14(1.54)	1.135	0.371–2.470	0.824	8(1.89)	6(1.41)	0.590
MI	10(2.21)	18(1.98)	0.643	0.275–1.504	0.309	9(2.12)	12(2.83)	0.507
CVA	15(3.31)	34(3.74)	0.597	0.191–1.873	0.377	14(3.30)	18(4.24)	0.471
Death/MI/CVA	29(6.40)	61(6.72)	0.636	0.354–1.144	0.131	27(6.37)	33(7.78)	0.422
Renal failure	19(4.19)	35(3.85)	1.076	0.419–2.763	0.880	18(4.24)	21(4.95)	0.623

MI, myocardial infarction; CVA, cerebrovascular accident; HR, hazard ratios; CI, confidence interval.

### Subgroup analyses

To evaluate the heterogeneity of the effects of PAC use with respect to in-hospital and 1-year mortality, we performed the subgroup analysis using age(<60 years vs.≥60 years), sex, LVEF(<50% vs.≥50%), and incidence of myocardial infarction. PAC use was not found to be a significant predictor for either high-risk or low-risk patients. (Tables 4 and 5).

**Table 4:** Subgroup analysis: The impact of pulmonary artery catheter use on in-hospital mortality.

In-hospital mortality	Control group(N = 908)		PAC group(N = 453)		Adjusted Odd ratio (95%CI)	P Value
	No. of patients	No. of events%	No. of patients	No. of events%		
Age group						
<60	444	4(0.9%)	163	2(1.2%)	1.374(0.241–7.840)	0.721
≥ 60	464	6(1.3%)	290	4(1.4%)	1.131(0.303–4.221)	0.854
Sex						
Female	198	2(1.0%)	122	2(1.6%)	1.026(0.132–7.960)	0.980
Male	710	8(1.1%)	331	4(1.2%)	1.221(0.353–4.228)	0.753
EF						
<50%	94	1(1.1%)	126	2(1.6%)	1.352(0.117–15.591)	0.809
≥ 50%	814	9(1.1%)	327	4(1.2%)	1.008(0.286–3.557)	0.990
MI status						
MI	249	2(0.8%)	254	3(1.2%)	1.376(0.220–8.610)	0.733
Non-MI	659	8(1.2%)	199	3(1.5%)	1.239(0.325–4.723)	0.754

**Table 5:** Subgroup analysis: The impact of pulmonary artery catheter use on 1-year mortality.

1-year mortality	Control group(N = 908)		PAC group(N = 453)		Hazard ratio (95%CI)	P Value
	No. of patients	No. of events%	No. of patients	No. of events%		
Age group						
<60	444	7(1.6%)	163	3(1.8%)	1.110(0.287–4.301)	0.879
≥ 60	464	7(1.5%)	290	5(1.7%)	0.994(0.314–3.149)	0.991
Sex						
Female	198	3(1.5%)	122	2(1.6%)	0.884(0.134–5.818)	0.898
Male	710	11(1.5%)	331	6(1.8%)	1.072(0.396–2.900)	0.891
EF						
<50%	94	3(3.2%)	126	3(2.4%)	0.772(0.155–3.839)	0.752
≥ 50%	814	11(1.4%)	327	5(1.5%)	1.039(0.359–3.009)	0.944
MI status						
MI	249	3(1.2%)	254	3(1.2%)	0.816(0.159–4.185)	0.807
Non-MI	659	11(1.7%)	199	5(2.5%)	1.497(0.520–4.309)	0.455

### Cost analyses

In-hospital costs are reported in Table 6. Cost for entire hospitalization was higher for PAC patients (mean, 87,211 vs. 83,240 RMB, respectively, approximately $14,535 vs. $13,873; p<0.001). There were no significant differences in separate preoperative,intraoperative, and postoperative costs (p>0.05). However, the preoperative, intraoperative and postoperative costs tended to be higher in PAC than control group (preoperative: mean, 11,492 vs. 10,712 RMB, approximately $1,915 vs. 1,785; p = 0.176; intraoperative: mean, 41,940 vs. 40,854 RMB, approximately $ 6,990 vs. 6,809; p = 0.068; postoperative: mean, 33,778 vs. 31,673 RMB, approximately $ 5,630 vs. 5,279; p = 0.057)

**Table 6:** In-hospital cost.

Variable	Entire cohort		P Value	Propensity-matched cohort		P Value
	PAC(N = 453)	Non-PAC(N = 908)		PAC(N = 424)	Non-PAC(N = 424)	
	Mean±SD	Mean±SD		Mean±SD	Mean±SD	
	RMB(USD)	RMB(USD)		RMB(USD)	RMB(USD)	
Entire cost	87211±19965	83240±25365	0.004	87112±19893	83117±25767	0.012
	(13821±3164)	(13192±4020)		(13805±3153)	(13172±4084)	
Preoperative cost	11492±8639	10712±10605	0.176	11415±8442	10598±10685	0.217
	(1821±1369)	(1698±1681)		(1809±1338)	(1680±1693)	
Intraoperative cost	41940±8583	40854±11080	0.068	41789±8207	40875±10889	0.168
	(6647±1360)	(6474±1756)		(6623±1301)	(6478±1726)	
Postoperaive cost	33778±14790	31673±21039	0.057	33908±14934	31642±21496	0.075
	(5353±2344)	(5019±3334)		(5374±2367)	(5015±3407)	

Costs are in Ren Min Bi (RMB). The exchange rate in 2012 was 6.31 RMB per US dollar.

## Discussion

Since it was introduced more than 40 years ago, substantial evidence has raised concern over PAC clinical use. It is clear that the PAC provides hemodynamic data that are used to make therapeutic decisions [10]. The critical question is whether these therapeutic decisions improve outcomes.

Shoemaker, et al [11] were the first to report on the use of hemodynamic data from PAC to determine fluid therapy and the use of vasoactive drugs. Several studies that evaluated PAC in the setting of CABG surgery have suggested that the benefits of PAC outweigh their risks in patients undergoing major cardiac and vascular surgery [12].

In contrast, debate regarding the efficacy and safety of PAC was rekindled by a multicenter observational study by Connors, et al [13] in 1996. The Understand Prognoses and Preferences for Outcomes and Risks of Treatments (SUPPORT) trial demonstrated higher mortality rates in patients who required PAC during hospitalization but no excess risk in patients with heart failure. After reports revealed that PAC increase mortality in patients with acute myocardial infarction, it is suggested that PAC use should be carefully considered. In the setting of CABG, a propensity-matched observational study [14] demonstrated that the use of a PAC during CABG surgery was associated with an increased mortality and a greater risk of severe end-organ complications.

However, several randomized trials in noncardiac surgery populations have reported no differences in mortality despite higher rates of catheter-related adverse events [3–5,15–17]. A meta-analysis of 13 trials including a total of 5,051 critically ill patients concluded that the PAC was associated with greater vascoactive drug use but had no impact on mortality or hospital length of stay [18]. In the setting of cardiac surgery, Tuman, et al [19] also identified the neutrality of the PAC for clinical outcomes.

While the ideal evaluation of the PAC in clinical practice would be a randomized controlled trial, such an undertaking is time-consuming, expensive, and of limited generalizability [20]. A statement by the American College of Chest Physicians (ACCP) and the American Thoracic Society (ATS) made in February of 1997 [21] recommended prospective, randomized, controlled trials on PAC use. Thereafter, only a few prospective randomized trials have been reported [3–5,15–17], and they have addressed PAC use in critically ill patients, high-risk surgical patients, shock and acute respiratory distress syndrome, congestive heart failure and acute lung injury. Further, these studies were limited by small sample sizes and lacked a strictly defined treatment protocol. It is difficult to design an RCT to assess PAC use because they are monitoring and diagnostic tools intended to guide clinical therapy. One such trial involved 226 patients undergoing CABG surgery [22], but it was also attributable to the small sample size and selection biases. There are no data from large, prospective, randomized studies to determine the impact of PAC use in CABG surgery [23]: CABG patients underwent unique hemodynamic and physiologic perturbations, and multiple therapeutic interventions.

This is the first and the largest study to investigate the relationships between PAC in CABG surgery and clinical and economic outcomes across China. After adjusting for several potentially confounding factors, our results indicated no differences in in-hospital or long-term outcomes between the CABG patients who were managed with or without a PAC, with the exceptions of significant greater use of intraoperative dopamine and epinephrine and administration among the PAC group. Subgroup analysis also identified neutrality of the PAC for clinical outcomes, irrespective of the degree preoperative LV function. Indeed, patients managed with the PAC incurred higher cost of initial hospitalization. The results were consistent with report by Person and colleagues [22] that no difference in outcomes between those given PAC and those managed by central venous catheterization. The total median costs in the PAC group were twice those in the non-PAC group.

Two organizations (the American Society of Anesthesiologists and the Society of Critical Care Medicine) [1,24] have developed guidelines for PAC application. These have been limited by broad generalizations. A survey has described current clinical practice attitudes among anesthesiologists in cardiac surgery in an effort to determine the most appropriate indications for PAC in Canada and the USA [20]. They identified that the two major predictors for appropriate use of PAC were impaired LV function and unstable angina. In our institution, administration of PAC was based on the anesthesiologist’s assessment. The question is not whether insertion of a PAC improves outcome but rather if PAC-derived data and therapy applied in accordance with these data by anesthesiologists improve outcomes, as integral parts of the overall monitoring and management of CABG patients. All the anesthesiologists in the study are trained to use PAC to ensure intensive hemodynamic manipulations and interventions as a result of the presence of a PAC. Given use or nonuse of PAC, anesthesiologists may consider different interpretation and treatment responses.

In our study, an increased use of vasoactive drugs in the patients who received PAC monitoring was observed. Tuman, el al [19] postulated that the greater use of agents in the PAC monitoring group might partly be a reflection of how monitoring and unnecessary information may affect therapy without significantly altering outcomes.

Thus, PAC should only be used when it is indicated. If a properly trained physician believes that invasive hemodynamic data are necessary to manage a specific patient, then the use of a PAC is justified. Our efforts should be concentrated on the training doctors to utilize PAC more wisely.

### Study limitations

This investigation was subject to several limitations. First, it was retrospective and performed at a single center. PAC use was not randomized and there were significant differences in covariates between the PAC and control groups before propensity score-matching. However, such a study design likely reduces confounding parameters and provides information complementary to earlier multi-center studies.Second, although our evaluation of the influence of PAC use was performed with score-matched groups, this evaluation might have underestimated the results because of potential confounding factors that were not investigated, such as social, educational, and personal. Third, all enrolled patients were ethnic Chinese; thus, the conclusions may apply only to Chinese or Asian populations. In one study that involved race-based utilization and outcomes of PAC [25], it was found that PAC use and in-hospital death were not determined by race. However, that study only compared the white and black, not an Asian population. Further large, well-designed and well-constructed, randomized, controlled trials might be required to give a more objective evaluation of PAC.

### Conclusion

This is the first and the largest study to investigate the relationships between pulmonary artery catheterization in CABG surgery and clinical and economic outcomes in China. Our findings provide no clear indication of any benefit or harm in managing CABG patients with PAC. However, using PAC in CABG is more expensive. That is, PAC use increased costs without benefit and thus appears unjustified for routine use in CABG surgery.

## Supporting Information

S1 Dataset All relevant data within the manuscript. (XLSX)
